# Association of Vitamin D Receptor and Vitamin D-Binding Protein Polymorphisms with Familial Breast Cancer Prognosis in a Mono-Institutional Cohort

**DOI:** 10.3390/nu13041208

**Published:** 2021-04-06

**Authors:** Valentina Aristarco, Harriet Johansson, Sara Gandini, Debora Macis, Cristina Zanzottera, Gianluca Tolva, Irene Feroce, Chiara Accornero, Bernardo Bonanni, Aliana Guerrieri-Gonzaga, Davide Serrano

**Affiliations:** 1 Division of Cancer Prevention and Genetics, European Institute of Oncology (IEO) IRCCS, 20141 Milan, Italy; harriet.johansson@ieo.it (H.J.); debora.macis@ieo.it (D.M.); cristina.zanzottera@ieo.it (C.Z.); gianluca.tolva@ieo.it (G.T.); irene.feroce@ieo.it (I.F.); chiaraarianna.accornero@ieo.it (C.A.); bernardo.bonanni@ieo.it (B.B.); aliana.guerrierigonzaga@ieo.it (A.G.-G.); davide.serrano@ieo.it (D.S.); 2 Department of Experimental Oncology, European Institute of Oncology (IEO) IRCCS, 20141 Milan, Italy; Sara.Gandini@ieo.it

**Keywords:** vitamin D, breast cancer, vitamin D receptor, vitamin D-binding protein

## Abstract

Low 25-hydroxyvitamin D (25OHD) has been associated with an increased cancer incidence and poorer prognosis. Single nucleotide polymorphisms (SNPs) of vitamin D receptor (VDR) and vitamin D binding protein (GC gene) may interfere with vitamin D activity. This study assesses the role of VDR and GC SNPs on breast cancer (BC) recurrence and survival in a cohort of patients with a family history of breast cancer, without the pathogenic variant for BRCA1 and BRCA2. A consecutive series of patients who underwent genetic testing were genotyped for VDR and GC genes. Specifically, ApaI, FokI, TaqI, BsmI and rs2282679, rs4588, rs7041 SNPs were determined. A total of 368 wild type (WT) patients with BC were analyzed for VDR and GC SNPs. The GC rs2282679 minor allele was significantly associated with luminal subtype of the primary tumor compared to Her2+/TN breast cancer (*p* = 0.007). Multivariate Cox models showed that BmsI and TaqI are significantly associated with BC outcome. Patients with the major alleles showed more than 30% lower hazard of relapse (BsmI *p* = 0.02 and TaqI *p* = 0.03). Our study supports the evidence for a pivotal role of 25OHD metabolism in BC. GC SNPs may influence the hormone tumor responsiveness and VDR may affect tumor prognosis.

## 1. Introduction

There is a growing body of literature that links an individual’s vitamin D status with their health condition. Low vitamin D has been correlated to several chronic diseases [1] including breast cancer risk and mortality. [2] The biologically active form, 1,25 dihydroxyvitamin D or calcitriol, binds a type II nuclear receptor (VDR), and may thereby regulate a variety of biological effects such as apoptosis, cell growth, adhesion, migration, metastases and angiogenesis [3,4]. The primary form of circulating vitamin D is 25 hydroxyvitamin D (25OHD), and it is considered to be a robust indicator of vitamin D status. In circulation, 25OHD is tightly bound to the vitamin D-binding protein (VDBP), encoded by the group-specific component gene (GC) [5]. Circulating 25OHD levels have been shown to be associated with tumor stage and survival, where lower levels were found in advanced stages of breast cancer and larger sized tumors, while higher levels were associated with a lower breast cancer risk in case-control studies [6,7], better prognosis in early breast cancer [8] and overall survival [9,10]. 

The role of vitamin D on cancer prognosis was confirmed by a meta-analysis of randomized clinical trials that showed a significant inverse association between vitamin D supplementation and cancer mortality [11].

The VDR gene is located on chromosome 12q12-14, and several single nucleotide polymorphisms (SNPs) have been investigated for cancer associations. The FokI SNP is associated with a shorter protein receptor, while BsmI (rs1544410), ApaI (rs7975232), and TaqI (rs731236), are located close to the 3′ terminus of the gene, and do not determine structural modifications of the protein [12]. There is no clear evidence about the effect of BsmI and ApaI variants in affecting the VDR activity, whereas TaqI is a synonymous SNP. Taq1 is in linkage disequilibrium with Bsm1 and Apa1, both located in the 3′-UTR region of the gene, thus outside the coding regions. These polymorphisms do not change the amino acid sequence of the protein, but they can affect gene expression, possibly through the control of mRNA stability [13,14]. These closely proximate polymorphisms are linked to another polymorphism, a variable-length poly-adenylate sequence (poly-A) within the 3′-UTR region. Depending on number of poly-A, the protein varies in length and may be segregated into two groups: long (18–24 A repeats) and short (13–17 A repeats). The TaqI SNP is located very close to the Poly A locus and may act as an indirect marker of Poly A variants [15,16]. 

The majority of cancers are sporadic. Hereditary breast cancer accounts for only 5–10% of all breast cancer cases. The tumor-suppressor genes BRCA1 and BRCA2 are the principal genes responsible for inherited cancer predisposition—40–80% of mutation carriers will develop a breast cancer in their life-time. However, the role of BRCA1 and BRCA2 mutation on prognosis remains controversial [17,18,19].

Among breast cancer patients meeting the criteria for genetic testing, in relation to hereditary breast and ovarian cancer syndrome, approximately one fourth are carriers of a pathogenic BRCA variant; thus, the majority of breast cancer patients are not BRCA mutation carriers defined as BRCA wild type (WT). We aimed to investigate whether SNPs in the VDR and GC gene may contribute to breast cancer relapse in patients with a family history of undefined origin.

## 2. Material and Methods

### 2.1. Study Participants

Study participants were recruited from the cohort of subjects assessed by genetic testing within the Division of Cancer Prevention and Genetics at the European Institute of Oncology. All subjects are included in a clinical database, containing general and tumor-related information, as well as clinical follow up such as relapse and/or death. All patients underwent genetic test in our institute but not all of them received primary surgery and histological confirmation in our institute.

The whole cohort included 1128 subjects who underwent genetic testing from 2002 to 2014.

Of this 1128, 657 were affected by breast cancer, 218 were BRCA1 or BRCA2 mutation carriers, 431 were WT and 8 were true negatives (relatives of known BRCA1 and BRCA2 mutation carriers negative for the known familial mutation and unaffected at the time of genetic testing) [20]. Out of the 431 WT breast cancers patients, we selected 368 women with invasive breast cancer. All women included in our analysis had a family history of breast and ovarian cancer. Each subject signed an informed consent approved by the Institutional Ethical Committee (IEO 1058). We excluded male subjects, carriers of pathogenic mutations in other disease-associated genes, people not affected at the time of genetic testing, and women with cancer in situ and with distant metastases at diagnoses.

**Figure 1:**
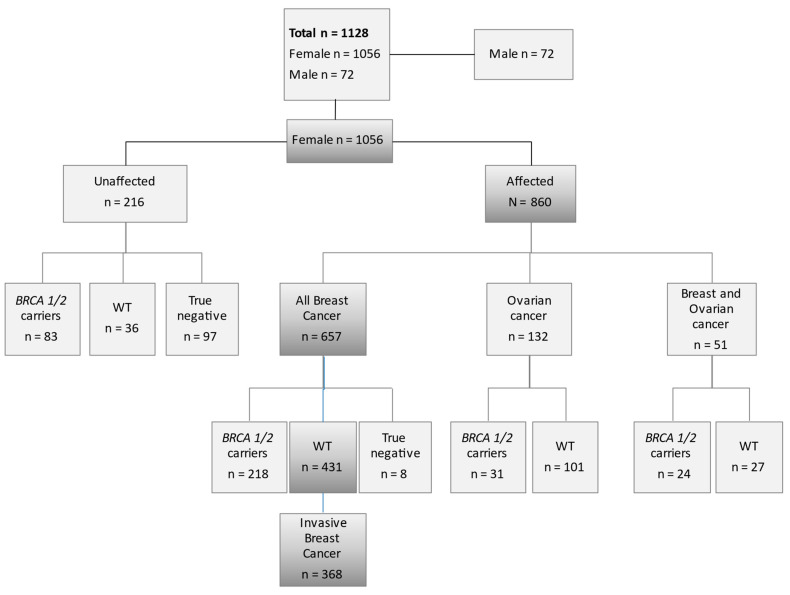
Flow diagram of patient selection. Shaded boxes trace the finial selected cohort. *n* = number of subjects; WT = wild type.

A participant flow diagram is shown in Figure 1.

Data on diagnosis and pathology were retrospectively retrieved from medical charts or directly from the pathologic report. Molecular tumor subtypes were classified according to 2011 St Gallen criteria [21]: briefly luminal-A: ER or PgR-positive and Ki-67 < 14% and HER2 negative; luminal-B: ER or PgR-positive and either Ki-67 ≥ 14% with negative HER2 or HER2-positive regardless of the Ki-67 value; HER2-positive and ER/PgR negative; and triple negative.

### 2.2. Genotype Analysis

Genomic DNA was extracted from whole blood specimens with a QIAamp DNA blood kit (Qiagen, Valencia, CA, USA) according to the manufacturer’s instructions by the automated platform “QIAcube” (Qiagen, Valencia, CA, USA) and quantified using NanoDrop spectrophotometer (Thermo Scientific, Wilmington, DE, USA). The germline DNA samples were genotyped for four SNPs: BsmI (rs1544410), TaqI (rs731236), FokI (rs228570), ApaI (rs7975232) in the VDR gene, and rs2282679, rs7041 and rs4588 in the GC gene.

SNPs genotyping was performed by the TaqMan SNP Genotyping Assays using an ABI PRISM 7500 FAST Real-Time PCR System (Thermo Fisher Scientific, Waltham, MA, USA) and a Sequence Detection Software ver.1.4 (Thermo Fisher Scientific, Waltham, MA, USA). Control samples, representing a complete set of genotypes for all SNPs, were processed in each run. Hardy–Weinberg equilibrium (HW) for genotype frequencies was tested using HW calculator (Michael H. Court, 2005–2008) downloaded from the internet.

### 2.3. Statistical Methods

Chi-squared or Fisher’s exact were used for categorical variables (e.g., to assess association between SNPs and histopathological features of breast cancers). 

Time to recurrence and to death were defined as the time from surgery until the event of interest. All patients alive or free of disease at the last follow-up date were considered right censored. Disease-Free Survival (DFS) and Overall Survival (OS) curves were estimated by the Kaplan–Meier method. The log-rank test was used to compare survival curves between groups. Cox proportional hazard models were used to identify independent predictors of survival/recurrence, with adjustment for relevant confounders and other prognostic factors.

All statistical tests were two-sided, and *p* < 0.05 was considered statistically significant. The statistical analyses were performed with the Statistical Analysis System Version 9.2 (SAS Institute, Cary, NC, USA).

## 3. Results

In our High Risk Patients’ Clinic, between 2002 and 2014, we counselled and tested 1128 subjects for BRCA1 and BRCA2 as shown in the flow diagram in Figure 1. Out of these 1128 subjects, we prospectively followed 368 women who had a diagnosis of invasive breast cancer, excluding subjects with intraepithelial neoplasia and WT for BRCA 1 and 2 genes. Baseline patients and tumor characteristics are reported in Table 1. Median age and Inter-Quartile Range (IQR) indicate a young population (42 years; IQR: 36–47). Median BMI and IQR indicate normal weight for the majority of the subjects (22 kg/m^2^; IQR: 20–25); 63% of women never smoked. All women had a family history of breast cancer and 9% also for ovarian cancer. Twenty-eight percent of cancers had lymph-node involvement and only 5% had a large tumor size (pT ≥ III), with 68% luminal subtypes. For 19% we lacked data to determine histopathological subtype. 

Table 2 shows the reference SNP numbers, minor allele frequencies and genotype frequencies for each analyzed SNP in the VDR and GC gene (BsmI, TaqI, FokI, ApaI; rs2282679, rs7041 and rs4588 respectively). All polymorphisms resulted in Hardy–Weinberg equilibrium. 

By comparing baseline tumor characteristics and GC SNPs (Table 3), we identified a statistically significant association between GC rs2282679 SNP and tumor molecular subtypes, with the minor allele associated with luminal A or B tumors (*p* = 0.007). No association was found between VDR SNPs and tumor characteristics. 

With nine years median follow-up from the primary surgery, there were 142 new events, in particular 33 ipsilateral and 36 contralateral relapses, 53 metastasis, 8 deaths and 12 other tumors.

**Figure 2:**
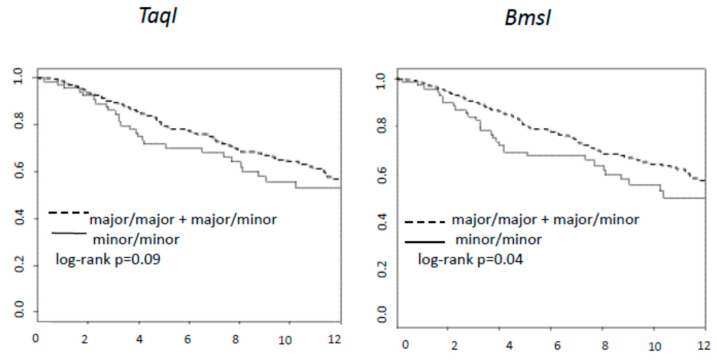
Kaplan and Meier curves of DFS and for BsmI and TaqI.

Figure 2 shows that subjects with minor allele homozygosis for BsmI and TaqI have the worst DFS (Log-rank tests for BsmI *p* = 0.04 and for TaqI *p* = 0.09). Multivariate analyses confirmed that the patients with at least one major allele for TaqI and BsmI showed a better prognosis compared to the homozygote minor alleles. The HR adjusted for pN and age estimate had more than 30% reduction of relapse; HR = 0.62 (95% CI 0.42–0.91) and HR = 0.64 (95% CI 0.43–0.96) for TaqI and BsmI, respectively (see Table 4).

No association was found between ApaI, BsmI, FokI, or GC SNPs and breast cancer DFS in univariate analyses and multivariate adjusted models. Polymorphisms were not found to be associated with overall survival.

## 4. Discussion

In this study we assessed the association of a comprehensive set of SNPs of VDR and GC genes and breast cancer prognosis in a cohort of 368 breast cancer patients with a positive family history of cancer but wild type for BRCA1 and BRCA2. Our findings support the hypothesis that genetic variants in the vitamin D pathway could have a role in breast cancer characteristics and outcome [22].

Vitamin D may influence breast cancer indirectly through its activity on the estrogen pathway [22]. Vitamin D signaling reduces estrogen-driven proliferation, maintains cell differentiation and downregulates estrogen receptor and aromatase expression [23,24,25]. 

The vitamin D binding protein polymorphisms are known to be associated with 25(OH)D level. In particular, rs2282679 and rs7041 have shown strong correlations with vitamin D levels [26,27]. In our population the GC rs2282679 SNP showed a significant correlation with tumor molecular subtype suggesting an interaction with the hormonal pathway and the minor allele of rs2282679 is associated with luminal A and B subtypes. More extensive analyses should be conducted to confirm the role of genetic variants of vitamin D metabolism in relation to breast cancer molecular subtypes. Clearly the determinants for the molecular subtype are not limited to one or few specific genes but can involve several genes. A recent large genome wide association study showed 32 new susceptibility variants for luminal and non-luminal molecular subtype of breast cancer [28]. Ultimately, polygenic risk scores could be developed to improve a personalized preventive medicine.

Since GC rs2282679 SNP correlate with lower level of circulating vitamin D, subjects with this genotype may not be protected from the hormonal down regulation seen in vitro [23,24,25]. Furthermore, as described in MCF7 tumor cell line and in a xenografts model, the breast cancer molecular subtype, particularly the different isoforms of Estrogen Receptor α may modulate by vitamin D signaling and in response to vitamin D supplementation [29]. VDR, in our cohort, did not show any correlation with tumor histology. A recent study by Kazemian et al. investigated the effects of biological interaction between VDR genetic polymorphisms and vitamin D supplementation in breast cancer survivors on biomarkers associated with inflammation, immune response but also cell proliferation, differentiation, damage and metastasis. They found that changes in certain inflammatory biomarkers in breast cancer survivors with low plasma 25(OH)D levels, supplemented with vitamin D3, may depend on VDR SNPs and haplotypes [30].

Our recent meta-analyses on VDR polymorphisms and cancer risk showed very heterogeneous results; in particular, TaqI and BsmI SNP and breast cancer did not show an overall significant correlation [31]. Data on vitamin D metabolism are more consistent on disease prognosis rather than cancer risk. Recently Jiang et al. analyzed several genetic variants in association with circulating 25(OH)D looking for a casual effect of vitamin D and various diseases. The results showed a casual effect for multiple sclerosis but limited effects on the other diseases including breast cancer [32]. However, a recent study supports the potential benefit of vitamin D on survival.

High nuclear VDR expression within tumor cells was associated with favorable prognostic factors and a decreased risk of breast cancer death [33]. A study from Perna et al. assessed the association of TaqI (rs731236) with breast cancer mortality. In a cohort of 498 BC patients followed for five years, they observed that the TaqI minor allele showed a trend toward an increased risk for breast cancer-specific mortality. The HR adjusted for age and breast cancer stage was 2.8 (95% CI 1.1–7.2) for breast cancer specify mortality and 2.1 (95% CI 0.9–4.9) for total mortality [34]. In our cohort we were able to show a significant correlation with disease free survival (HR 0.64 for TaqI), but not for overall survival. 

A study based on Pakistani women [35] showed an association with BsmI B allele and breast cancer risk, whereas a study looking at breast cancer susceptibility in an Iranian population [36] showed a significant association between BsmI b allele and increased risk of breast cancer. In both studies the association was significant overall, maintained in subjects with no BRCA 1 and 2 mutations, but the significance was lost in the BRCA mutation carriers. Our study was based on a Caucasian population, looking at recurrences rather than incidence and BsmI major (b) allele showed a correlation with a better prognosis (HR 0.62). 

The strength of the study lies in the long follow-up period and the homogeneity of the selected population, in terms of prognostic factors and positive family history with *BRCA* 1/2 WT. One limitation is that since the time of blood collection was extremely variable among the participants from the breast cancer diagnosis, it would not be reasonable to look at circulating biomarkers such as 25(OH)D or vitamin D binding protein levels. A second limitation is a relatively small sample size, thus we could not adjust for multiple testing because of low statistical power. However, no formal statistical hypotheses were pre-specified to determine the sample size since this is an observational exploratory study and results will have to be validated in a further larger cohort study.

## 5. Conclusions

Our analysis supports the hypothesis that 25(OH)D metabolisms have a role in breast cancer prognosis. VDBP polymorphisms may have an influence through estrogen signaling of the molecular subtype tumor characteristic. VDR polymorphisms and in particular BsmI and TaqI may influence cancer outcome. Overall, vitamin D should be considered to address cancer risk and prognosis in order to enhance the opportunity for breast cancer prevention especially in high risk cohorts with a positive family history. Further prospective studies should be conducted to confirm these hypotheses and to assess the potential beneficial effect of vitamin D supplementation in the context of primary and tertiary cancer preventive medicine. 

## Figures and Tables

**Table 1:** Baseline patients and tumor characteristics of breast cancer women wild type for BRCA1/2.

**Patients Characteristics**		***n***	**%**
		368	100
Age at diagnosis, y: Median (Q1–Q3)	41 (36–47)		
BMI, kg/m^2^: Median (Q1–Q3)	22.3 (20.5–24.9)		
Age at menarche, y: Median (Q1–Q3)	12 (11–13)		
Oral contraceptive	No	139	36.8
	Yes	225	59.5
	Missing	14	3.7
Parity	No	99	26.9
	Yes	269	73.1
Family history	Breast	335	91.1
	Breast and ovary	33	8.9
Personal history of other cancer	No	355	96.5
	Yes	13	3.5
Smoking	Former	98	26.6
	No	231	62.8
	Current	39	10.6
Molecular subtype	Luminal A	75	20.4
	Luminal B	141	38.3
	Luminal B-Her2+	36	9.8
	HER2+	25	6.8
	Triple negative	22	6.0
	Missing	69	18.8
pT	I	246	66.9
	II	86	23.4
	III	18	4.9
	IV	1	0.3
	Missing	18	4.6
pN	Negative	246	66.8
	Positive	86	28.3
	Missing	18	4.9

Abbreviations: WT = Wild Type for BRCA1/2; BMI = Body Mass Index; VDBP = vitamin D binding protein; pT = pathological stage; pN = pathological assessment of lymph-node involvement; Q1–Q3 = I and III quartile.

**Table 2:** Reference SNP numbers, minor allele frequencies (MAFs) and genotype.

Gene	SNP	*n*	MAF	Genotype, *n* %			
				Major/Major	Major/Minor	Minor/Minor	HWE *p* Value
*VDR*	rs1544410 BsmI (C > T)	368	0.45 (T)	113 (31)	185 (50)	70 (19)	0.85
*VDR*	rs2228570 FokI (A > G)	368	0.40 (A)	147 (40)	179 (49)	42 (11)	0.76
*VDR*	rs731236 TaqI (A > G)	368	0.45 (G)	119 (32)	184 (50)	65 (18)	0.88
*VDR*	rs7975232 ApaI (A > C)	368	0.42 (C)	132(36)	176 (48)	60 (16)	0.08
*GC*	rs2282679 (T > G)	368	0.26 (G)	182 (49)	155 (42)	31 (8)	0.97
*GC*	rs4588 (G > T)	368	0.26 (T)	158 (43)	157 (43)	53 (14)	0.87
*GC*	rs7041 (A > C)	368	0.59 (C)	71 (20)	180 (49)	117 (32)	0.38

Abbreviations: VDR, vitamin D receptor; GC, Globulin Complex; SNP, single nucleotide polymorphism; MAF, minor allele frequency (frequency at which the second most common allele occurs in the population); HWE, Hardy-Weinberg Equilibrium.

**Table 3:** GC SNPs variants frequencies by breast cancer molecular subtypes.

SNP			Major/Major	Major/Minor	Minor/Minor	Total	*p* Value
	Histotype	Total	*n* = 182	*n* = 155	*n* = 31	*n* = 368	
GC rs228679	Luminal A or B	*n*.	115	117	20	252	
		%	63.19	75.48	64.5	83.43	
	HER2+ or TN	*n*.	33	13	1	47	0.007
		%	18.13	8.39	3.23	12.77	
	Missing	*n*.	34	25	10	69	
		%	18.68	16.13	32.26	18.75	
		Total	*n* = 158	*n* = 157	*n* = 53	*n* = 368	
GC rs4588	Luminal A or B	*n*.	37	34	12	83	
		%	23.42	21.66	22.64	22.55	
	HER2+ or TN	*n*.	87	98	31	216	0.766
		%	55.06	62.42	58.49	58.70	
	Missing	*n*.	34	25	10	69	
		%	21.52	15.92	18.87	18.75	
		Total	*n* = 71	*n* = 180	*n* = 117	*n* = 368	
GC rs7041	Luminal A or B	*n*.	12	38	33	83	
		%	16.90	21.11	28.21	22.55	
	HER2+ or TN	*n*.	43	114	59	216	0.102
		%	60.56	63.33	50.43	58.70	
	Missing	*n*.	16	28	25	69	
		%	22.54	15.56	21.37	18.75	

*p* value calculated without missing values. HER2+ = human epidermal growth factor receptor 2 positive breast cancer; TN= triple negative breast cancer.

**Table 4:** Results from Multivariate Cox Proportional hazard model for disease-free survival and significant VDR.

		HR	Low 95%CI	Up 95%CI	*p*-Values
Age		0.98	0.96	1.00	0.08
pN+	No vs. Yes	0.51	0.36	0.72	<0.001
*TaqI*	Major/Major Major/Minor vs Minor/Minor	0.64	0.43	0.96	0.03
Age		0.98	0.96	1.00	0.07
pN+	No vs Yes	0.50	0.36	0.72	<0.001
*BsmI*	Major/Major Major/Minor vs Minor/Minor	0.62	0.42	0.91	0.02

pN+: Lymph node status: no = negative, yes = positive.

## Data Availability

The data underlying this article will beshared on reasonable request to the head of the Division of Cancer Prevention and Genetics Dr Bernardo Bonanni.

## References

[1] Muscogiuri G. (2018). Vitamin D: Past, present and future perspectives in the prevention of chronic diseases. Eur. J. Clin. Nutr..

[2] Estébanez N., Gòmez-Acebo I., Palazuelos C., Llorca J., Dierssen-Sotos T. (2018). Vitamin D exposure and Risk of Breast Cancer: A meta-analysis. Sci. Rep..

[3] Welsh J. (2007). Targets of vitamin D receptor signaling in the mammary gland. J. Bone Miner Res..

[4] Artaza J.N., Sirad F., Ferrini M.G., Norris K.C. (2010). 1, 25(OH)2vitamin D3 inhibits cell proliferation by promoting cell cycle arrest without inducing apoptosis and modifies cell morphology of mesenchymal multipotent cells. J. Steroid Biochem. Mol. Biol..

[5] Delanghe J.R., Speeckaert R., Speeckaert M.M. (2015). Behind the scenes of vitamin D binding protein: More than vitamin D binding. Best Pract. Res. Clin. Endocrinol. Metab..

[6] Garland C.F., Gorham E.D., Mohr S.B., Grant W.B., Giovannucci E.L., Lipkin M. (2007). Vitamin D and prevention of breast cancer: Pooled analysis. J. Steroid Biochem. Mol. Biol..

[7] Abbas S., Linseisen J., Slanger T., Kropp S., Mutschelknauss E.J., Flesch-Janys D., Chang-Claude J. (2008). Serum 25-hydroxyvitamin D and risk of post-menopausal breast cancer—results of a large case-control study. Carcinogenesis.

[8] Goodwin P.J., Ennis M., Pritchard K.I., Koo J., Hood N. (2009). Prognostic effects of 25-hydroxyvitamin D levels in early breast cancer. J. Clin. Oncol..

[9] Villaseñor A., Ballard-Barbash R., Ambs A., Bernstein L., Baumgartner K., Baumgartner R. (2013). Associations of serum 25-hydroxyvitamin D with overall and breast cancer-specific mortality in a multiethnic cohort of breast cancer survivors. Cancer Causes Control.

[10] Yao S., Kwan M.L., Ergas I.J., Roh J.M., Cheng T.Y.D., Hong C.C. (2017). Association of Serum Level of Vitamin D at Diagnosis With Breast Cancer Survival: A Case-Cohort Analysis in the Pathways Study. JAMA Oncol..

[11] Keum N., Lee D.H., Greenwood D.C., Manson J.E., Giovannucci E. (2019). Vitamin D supplementation and total cancer incidence and mortality: A meta-analysis of randomized controlled trials. Ann. Oncol..

[12] Uitterlinden A.G., Fang Y., van Meurs J.B., Pols H.A., van Leeuwen J.P. (2004). Genetics and biology of vitamin D receptor polymorphisms. Gene.

[13] Durrin L.K., Haile R.W., Ingles S.A., Coetzee G.A. (1999). Vitamin D receptor 3’-untranslated region polymorphisms: Lack of effect on mRNA stability. Biochim. Biophys. Acta.

[14] Whitfield G.K., Remus L.S., Jurutka P.W., Zitzer H., Oza A.K., Dang H.T. (2001). Functionally relevant polymorphisms in the human nuclear vitamin D receptor gene. Mol. Cell Endocrinol..

[15] Guy M., Lowe L.C., Bretherton-Watt D., Mansi J.L., Peckitt C., Bliss J. (2004). Vitamin D receptor gene polymorphisms and breast cancer risk. Clin. Cancer Res..

[16] Kostner K., Denzer N., Muller C.S., Klein R., Tilgen W., Reichrath J. (2009). The relevance of vitamin D receptor (VDR) gene polymorphisms for cancer: A review of the literature. Anticancer Res..

[17] Easton D.F. (1999). How many more breast cancer predisposition genes are there?. Breast Cancer Res..

[18] Campeau P.M., Foulkes W.D., Tischkowitz M.D. (2008). Hereditary breast cancer: New genetic developments, new therapeutic avenues. Hum. Genet..

[19] Antoniou A., Pharoah P.D., Narod S. (2003). Average risks of breast and ovarian cancer associated with BRCA1 or BRCA2 mutations detected in case Series unselected for family history: A combined analysis of 22 studies. Am. J. Hum. Genet..

[20] Domchek S.M., Gaudet M.M., Stopfer J.E., Fleischaut M.H., Powers J., Kauff N. (2010). Breast cancer risks in individuals testing negative for a known family mutation in BRCA1 or BRCA2. Breast Cancer Res. Treat..

[21] Goldhirsch A., Wood W.C., Coates A.S., Gelber R.D., Thurlimann B., Senn H.J. (2011). Strategies for subtypes--dealing with the diversity of breast cancer: Highlights of the St. Gallen International Expert Consensus on the Primary Therapy of Early Breast Cancer 2011. Ann. Oncol..

[22] Vaughan-Shaw P.G., O’sullivan F., Farrington S., Theodoratou E., Campbell H., Dunlop M.G., Zgaga L. (2017). The impact of vitamin D pathway genetic variation and circulating 25-hydroxyvitamin D on cancer outcome: Systematic review and meta-analysis. Br. J. Cancer.

[23] Krishnan A.V., Swami S., Peng L., Wang J., Moreno J., Feldman D. (2010). Tissue-selective regulation of aromatase expression by calcitriol: Implications for breast cancer therapy. Endocrinology.

[24] Krishnan A.V., Swami S., Feldman D. (2010). Vitamin D and breast cancer: Inhibition of estrogen synthesis and signaling. J. Steroid Biochem. Mol. Biol..

[25] Swami S., Krishnan A.V., Peng L., Lundqvist J., Feldman D. (2013). Transrepression of the estrogen receptor promoter by calcitriol in human breast cancer cells via two negative vitamin D response elements. Endocr. Relat. Cancer.

[26] Wang T.J., Zhang F., Richards J.B., Kestenbaum B., Van Meurs J.B., Berry D. (2010). Common genetic determinants of vitamin D insufficiency: A genome-wide association study. Lancet.

[27] Santos B.R., Mascarenhas L.P., Boguszewski M.C., Spritzer P.M. (2013). Variations in the vitamin D-binding protein (DBP) gene are related to lower 25-hydroxyvitamin D levels in healthy girls: A cross-sectional study. Horm. Res. Paediatr..

[28] Zhang H., Ahearn T.U., Lecarpentier J., Barnes D., Beesley J., Qi G. (2020). Genome-wide association study identifies 32 novel breast cancer susceptibility loci from overall and subtype-specific analyses. Nat. Genet.

[29] Verma A., Schwartz Z., Boyan B.D. (2019). 24R, 25-dihydroxyvitamin D(3) modulates tumorigenicity in breast cancer in an estrogen receptor-dependent manner. Steroids.

[30] Kazemian E., Akbari M.E., Moradi N., Gharibzadeh S., Mondul A.M., Jamshidi-Naeini Y., Khademolmele M., Zarins K.R., Ghodoosi N., Amouzegar A. (2019). Vitamin D Receptor Genetic Variation and Cancer Biomarkers among Breast Cancer Patients Supplemented with Vitamin D3: A Single-Arm Non-Randomized Before and After Trial. Nutrients.

[31] Gnagnarella P., Raimondi S., Aristarco V. (2020). Vitamin D Receptor Polymorphisms and Cancer. Sunlight, Vitamin D and Skin Cancer.

[32] Jiang X., Ge T., Chen C.Y. (2021). The causal role of circulating vitamin D concentrations in human complex traits and diseases: A large-scale Mendelian randomization study. Sci. Rep..

[33] Huss L., Butt S.T., Borgquist S. (2019). Vitamin D receptor expression in invasive breast tumors and breast cancer survival. Breast Cancer Res..

[34] Perna L., Butterbach K., Haug U., Schöttker B., Müller H., Arndt V. (2013). Vitamin D receptor genotype rs731236 (Taq1) and breast cancer prognosis. Cancer Epidemiol. Biomark. Prev..

[35] Rashid M.U., Muzaffar M., Khan F.A., Kabisch M., Muhammad N., Faiz S. (2015). Association between the BsmI Polymorphism in the Vitamin D Receptor Gene and Breast Cancer Risk: Results from a Pakistani Case-Control Study. PLoS ONE.

[36] Shahabi A., Alipour M., Safiri H., Tavakol P., Alizadeh M., Hashemi S.M., Shahabi M., Halimi M. (2017). Vitamin D Receptor Gene Polymorphism: Association with Susceptibility to Early-Onset Breast Cancer in Iranian, BRCA1/2-Mutation Carrier and non-carrier Patients. Pathol. Oncol. Res..

